# A New Approach for Advertising CTR Prediction Based on Deep Neural Network via Attention Mechanism

**DOI:** 10.1155/2018/8056541

**Published:** 2018-09-13

**Authors:** Qianqian Wang, Fang'ai Liu, Shuning Xing, Xiaohui Zhao

**Affiliations:** ^1^School of Information Science and Engineering, Shandong Normal University, Jinan, China; ^2^School of Mathematical Science, Shandong Normal University, Jinan, China

## Abstract

Click-through rate prediction is critical in Internet advertising and affects web publisher's profits and advertiser's payment. The traditional method of obtaining features using feature extraction did not consider the sparseness of advertising data and the highly nonlinear association between features. To reduce the sparseness of data and to mine the hidden features in advertising data, a method that learns the sparse features is proposed. Our method exploits dimension reduction based on decomposition, takes advantage of the attention mechanism in neural network modelling, and improves FM to make feature interactions contribute differently to the prediction. We utilize stack autoencoder to explore high-order feature interactions and use improved FM for low-order feature interactions to portray the nonlinear associated relationship of features. The experiment shows that our method improves the effect of CTR prediction and produces economic benefits in Internet advertising.

## 1. Introduction

Click-through rate (CTR) prediction is critical to many web applications including web search, recommender systems [1, 2], sponsored search, and display advertising. Search advertising, known as sponsored search, refers to advertisers identifying relevant keywords based on their product or service for advertising. When the user retrieves the keyword purchased by the advertiser, the corresponding advertisement is triggered and displayed. In the cost-per-click model, the advertiser pays the web publisher only when a user clicks their advertisements and visits the advertiser's site. The CTR prediction is defined to estimate the ratio of clicks to impressions of advertisements that will be displayed [3].

With the rapid development of the mobile Internet and its wide range of applications, advertising has become one of the most successful business models in the world. Internet text advertising is regarded as a more effective advertising communication method due to its strong targeted communication and convenience of user clicking and has become an important income resource for many Internet companies. Some electronic commerce companies and search engine companies are seeking targeted advertising to increase their revenue.

In general, the display of online advertising can be seen as a three-party game between media, advertisers, and users. How to advertise to specific user groups is a key issue in the field of online advertising. Inappropriate advertising can lead to a decline in user experience. Advertising cannot achieve the desired effect, and the media can also be affected. Internet text advertising is usually in the form of text, and the advertisers get the opportunity to buy media ads through cost-per-click (CPC) [4]. In the CPC model, the click-through rate (CTR) is an important indicator to measure the effectiveness of advertising display and is a key factor in the three-party game. Therefore, the CTR estimation of advertising is a hot research direction in the field of computing advertising. In this paper, the click-through rate prediction of Internet text advertising shows the probability of predicting a user's click on a text under the current context environment. Due to the three-party information of advertising properties, user properties, and context environment, the CTR prediction is very complicated.

At present, the prediction of click-through rate for online advertising has attracted widespread attention from researchers in industry and academia. Researchers have proposed many models that are usually based on machine learning methods. We can divide them into three categories: linear, nonlinear, and fusion models. Typically, a predictive task is formulated as estimating a function that maps predictor variables to some target. To build predictive models with these predictor variables, a common solution is to convert them to a set of binary features (a.k.a. feature vector) via one-hot encoding [5]. McMahan et al. [6] used the logistic regression [7] model to solve the CTR problems of Google Advertising. They adopted user information, advertising data, search keywords, and other features as the input of the model and proposed an online sparse learning algorithm to train the model. Chapelle [8] proposed a machine-learning framework based on the logistic regression in which advertisers, web publishers, users, and time characteristics were used as input to the model to solve the advertising CTR prediction for Yahoo. Dave and Varma [9] used the gradient boosting decision tree (GBDT) to predict the advertising CTR. They extracted similar features from advertising data and discovered implicit relationships between different features. Finally, they found out the nonlinear relationships between the predicted target and features. He et al. [10] introduced a fusion model which combines decision trees with logistic regression for predicting clicks on Facebook ads. The traditional CTR prediction model mainly depends on the design of features. The features of data are artificially selected and processed. The data have a complex mapping relationship, especially for meaningful data, and it is crucial to account for the interactions between features. Many successful solutions in both industry and academia largely rely on manually crafting combinatorial features [11], i.e., constructing new features by combining multiple predictor variables, also known as cross features. However, the power of such features comes at a high cost since it requires heavy engineering efforts and useful domain knowledge to design effective features. Factorization machines (FMs) [12] are a supervised learning approach that embed features into a latent space and model the interactions between features via inner product of their embedding vectors. Models based on degree-2 polynomial mapping and factorization machines are widely used for CTR prediction. The factorization-based prediction method field-aware factorization machines [13] were developed by Juan et al.

In recent years, deep learning [14, 15] has achieved very good results in the fields of speech recognition [16], image data processing [17], and natural language processing [18]. As a powerful approach to learning feature representation, deep neural networks have the potential to learn sophisticated feature interactions. Liu et al. [19] extended CNN for CTR prediction, but CNN-based models are biased towards the interactions between neighboring features. Zhang et al. [20] studied feature representations and proposed factorization machine-supported neural network (FNN). This model pretrained FM before applying DNN and thus limited by the capability of FM. He and Chua [21] proposed a novel neural factorization machine (NFM) for prediction under sparse setting. NFM combines the linearity of FM in modelling second-order feature interactions and the nonlinearity of neural network in modelling higher-order feature interactions. Despite great promise, we argue that FM can be hindered by its modelling of all factorized interactions with the same weight. In real-world applications, different predictor variables often have different predictive power. Not all features contain useful information for predicting the target. Therefore, the interaction of features with less useful information should be assigned a lower weight indicating that they contribute less to the prediction. However, FM lacks the ability to distinguish the importance of feature interactions, which will lead to suboptimal prediction.

Considering the high-dimensional sparsity of advertising data and the highly nonlinear association between features [22], a hybrid model for advertising CTR estimation based on stacked autoencoder, named Attention Stacked Autoencoder (ASAE), is proposed. Our model takes advantage of the attention mechanism in neural network modelling [23, 24] and improves FM to make feature interactions contribute differently to the prediction. More importantly, the importance of feature interactions is automatically learned from the data with any human domain knowledge. We explore data dimension reduction and identify the relationship between features. Additionally, many experiments are conducted to show that this method improves the accuracy of CTR estimation.

The rest of this paper is organized as follows.  Section 2 provides the factorization machines. In Section 3, the sparse feature learning method for advertising data based on the ASAE model is proposed. In Section 4, we design the experiment and verify the prediction effect of the method by comparison experiment. We also analyze the experimental results in this section.  Section 5 concludes the paper and lists possible future work.

## 2. Factorization Machines

The factorization machines are originally proposed for learning feature interactions in the recommendation system. Given a real-valued feature vector *X* ∈ *ℝ* ^*n*^ where *n* denotes the number of features, FM estimates the target by modelling all interactions between each pair of features:

(1) $$\begin{array}{c} \hat{y}\text{FM}\left(x\right)=w_{0}+\overset{n}{\underset{i=1}{\sum }}w_{i}x_{i}+\overset{n}{\underset{i=1}{\sum }}\overset{n}{\underset{j=i+1}{\sum }}{\hat{w}}_{ij}x_{i}x_{j}, \end{array}$$

where *w*_0_ is the global bias, *w*_*i*_ denotes the weight of the *i*th feature, and *w*_*ij*_ denotes the weight of the cross feature *x*_*i*_ *x*_*j*_, which is factorized as ${\hat{w}}_{ij}=v_{i}^{T}v_{j}$, where *v*_*i*_ ∈ *ℝ* ^*k*^ denotes the embedding vector for feature *i* and *k* denotes the size of the embedding vector. Besides linear (order-1) interactions among features, FM models pairwise (order-2) feature interactions as inner product of respective feature latent vectors. It can capture order-2 feature interactions much more effectively than previous approaches especially when the dataset is sparse. It is worth noting that FM models all feature interactions in the same way: first, a latent vector *v*_*i*_ is shared in estimating all feature interactions that the *i*th feature involves; second, all feature interactions have the same weight of 1. However, it is common that not all features are relevant to the prediction. These interactions of irrelevant features can be considered as noise that does not contribute to the prediction. FM models all features using the same weights for interaction and may have a negative impact on generalization performance.

## 3. Click-through Rate Estimation Based on Deep Neural Network

One of the necessary steps in the click rate prediction system is to mine features that are highly correlated with the estimated task. To reduce the high sparseness of features and characterize the nonlinear association between features, we propose a sparse feature learning method for advertising data based on deep learning (DLSAE).

### 3.1. Data Dimensionality Reduction

Click log data contain many types of objects, such as users, queries, and advertisements. The relationship between these objects is very complex. The same objects have similarity, and there are complex relationships between different types of objects. For instance, given a particular user and the query submitted by the user, it is necessary to predict whether the user will click on the advertisement and the probability. There is a complex implicit relationship between users, queries, and advertising. Based on the characteristics of the click log data, dimension reduction is achieved in the following two aspects: the similarity between the internal objects and the association between different objects.

In this paper, the k-means clustering algorithm [25] based on distance is adopted. We cluster queries, advertisements, and users separately, and the similar objects are aggregated into the same cluster. We use advertising frequency as the weight of the advertisement *A*_*i*_ and query *Q*_*j*_ and create a matrix *W*_*M*_a_×*M*_q__ of the ad-query (where *M*_a_ is the number of ads and *M*_q_ indicates the number of queries), using the k-means algorithm to cluster the ad-query matrix. We scan the ad-query matrix to obtain the ad sets and query sets, as *A*={*a*_1_, *a*_2_,…, *a*_*m*_} and *Q*={*q*_1_, *q*_2_,…, *q*_*N*_}. Then, we take *K* samples from the advertising set randomly as the initial point of the cluster center, record as *T*={*t*_1_, *t*_2_,…, *t*_*k*_}. Next, Equation (2) is used to calculate the distance between ad *a*_*i*_ and each cluster center point *t*_*j*_. The number of clusters of users, ads, and queries is represented by *K*_u_, *K*_a_, and  *K*_q_, respectively. Finally, the number of users, ads, and queries in the dataset is reduced from *M*_u_, *M*_a_, *M*_q_ to *K*_u_, *K*_a_, *K*_q_:

(2) $$\begin{array}{c} \text{Dis}\left(a_{i},t_{j}\right)=\sqrt{\sum {\left(W_{a_{i}}-W_{q_{j}}\right)}^{2}}, \end{array}$$

where *W*_*a*_*i*__ is the weight of *a*_*i*_, *W*_*q*_*j*__ is the weight of *t*_*j*_, and Dis(*a*_*i*_, *t*_*j*_) is the distance between *a*_*i*_ and *t*_*j*_.

There is a ternary relationship between the user-query-ad in the click log data. In this paper, we use the three-dimensional tensor structure model [26, 27] to represent the user, query, and advertisement. Then, the tensor decomposition method is used to reduce the dimensions. The sum of the display number of ads in the cluster is used as the weight of the elements in 3D space. The three-dimensional tensor model is constructed and represented by *X* ∈ *R* ^*K*_u_×*K*_q_×*K*_a_^. In this paper, tensor *X* is decomposed using the Tucker factorization. Equation (2) is the decomposition formula.

(3) X=G;A,B,C=G×Au×Bq×Ca=∑p=1P∑m=1M∑r=1Rgpmrup ∘ qm ∘ ar,

where *G* represents the core tensor of tensor *X*. We use *A*, *B*, and *C* to represent the feature matrix of the tensor *X* on the dimension *K*_u_, *K*_q_, *K*_a_.

**Figure 1:**
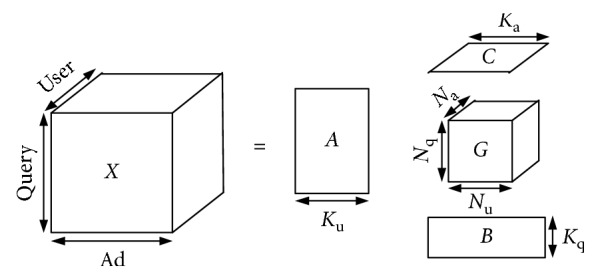
Schematic diagram of the Tucker decomposition.

Figure 1 is a schematic diagram of the Tucker decomposition. The purpose of the Tucker decomposition is to find an approximate tensor $\hat{X}$ with the original tensor *X* and to retain the original tensor information and structural information to the greatest extent. The minimization formula is shown below:

(4) minX−X^, X^=G×Au×Bq×Ca=G;A,B,C.

Equation (4) is the objective optimization function. According to Equation (3), the expression of the core tensor can be obtained as follows:

(5) G=X×AuT×BqT×CaT,

and the objective function can be written in a squared form:

(6) X−G;A,B,C2=X2−2X×AuT×BqT×CaT,G+G2,=X2−2G,G+G2,=X2−G2,=X2−X×AuT×BqT×CaT2.

Therefore, the objective function is transformed to

(7) maxX×AuT×BqT×CaT2,ATW, W=X×BqT×CaT,BTW, W=X×AuT×CaT,CTW, W=X×AuT×BqT.

In the process of solving the optimal solution, we need to fix the matrix of the other dimensions *W*, solve for *A* ^*Τ*^, *B* ^*Τ*^, *C* ^*Τ*^, and then perform a singular value decomposition (SVD) of *A* ^*Τ*^, *B* ^*Τ*^, *C* ^*Τ*^. Next, expand the tensor *X* into a matrix on the user, query, and advertising dimensions, respectively, as *X*_1_, *X*_2_, *X*_3_ and apply SVD on *X*_1_, *X*_2_, *X*_3_:

(8) $$\begin{array}{c} X_{1}=A\cdot G_{1}\cdot V_{1}^{T},\\ X_{2}=B\cdot G_{2}\cdot V_{2}^{T},\\ X_{3}=C\cdot G_{3}\cdot V_{3}^{T}, \end{array}$$

where *G*_1_, *G*_2_, *G*_3_ are the diagonal singular value matrices obtained using singular value decomposition of the matrices *X*_1_, *X*_2_, *X*_3_. *g*_1_, *g*_2_, *g*_3_ are the dimensions of the singular value matrix *A*, *B*, *C*. The dimensions *g*_1_, *g*_2_, *g*_3_ are obtained by calculating the diagonal singular values of *G*_1_, *G*_2_, *G*_3_ in proportion. In the process of reducing the dimensions, the proportion of excluded singular values is set to 50% in this paper. Therefore, the calculation of the core tensor after dimension reduction is as follows:

(9) G′=X×Aur1T×Bqr2T×Car3T,X′=G′×Aur1×Bqr2×Car3.

The three dimensions of the initial tensor *X* are *K*_u_, *K*_q_, *K*_a_, and the three dimensions of the approximate tensor *X*′ after decreasing dimension are denoted by *N*_u_, *N*_q_, *N*_a_. The time complexity of the Tucker decomposition algorithm is proportional to the tensor dimension, which is expressed as *O*(*K*_u_ *K*_q_ *K*_a_). We previously used the clustering method to achieve the reduction of the original matrix, which reduced the cost of the Tucker decomposition greatly and improved the efficiency and precision.

### 3.2. Feature Composition Analysis of the Input Layer

**Figure 2:**
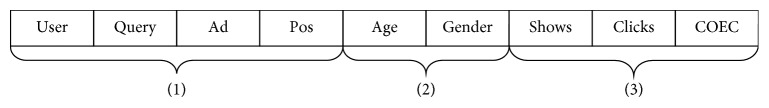
The features of the input layer.

There is a high degree of nonlinear correlation between the features in advertising data. Although the approximate tensor of the original tensor is reduced by the Tucker decomposition, it only reflects the information between the three characteristic dimensions of user, query, and ad. Other useful information in the data is not fully utilized for click-through rate estimates, such as the position of the advertisement on the page, the number of ads, and the age and gender of the user. This paper combines the features of <user, query, ad> after tensor reduction and other valid information in the log data as the object of feature learning. The composition of the input layer features is summarized as follows:

1. *ID Feature*. ID feature uniquely identifies a class of entities in the actual click log, usually using a set of numeric strings to represent variables. For instance, “10110” can identify only one user group. The ID class used in this article has the UserID, QueryID, AdID, position, and the number of advertisements on the return page. UserID, QueryID, and AdID are collections of “virtual” ID classes that are obtained using k-means clustering and tensor dimension reduction.
2. *Attribute Characteristics*. The ID class feature is a symbol that cannot be obtained from the new entity data and has weak generalization ability. Attribute features are used to describe a set of users, ad collections, etc., and have better generalization ability and apply to multiple instances. Therefore, it is necessary to attribute the property class as learning the input layer feature further. Commonly used attribute class features are user's URL, user's gender, user's age, and advertising time to trigger and query keywords.
3. *Statistical Characteristics*. The statistical feature uses historical data statistics information to provide an estimate for the forecasting model. The statistical characteristics of the text consist of the number of advertising histories, the number of clicks on the advertising history, and the click-through rate after the advertising position normalization, denoted by Shows, Clicks, and COEC In the experiment, the input layer feature of the ASAE model is shown in Figure 2.

### 3.3. Study on CTR Prediction via Attention Mechanism Based on the Stacked Autoencoder

#### 3.3.1. Attentional Factorization Machines

**Figure 3:**
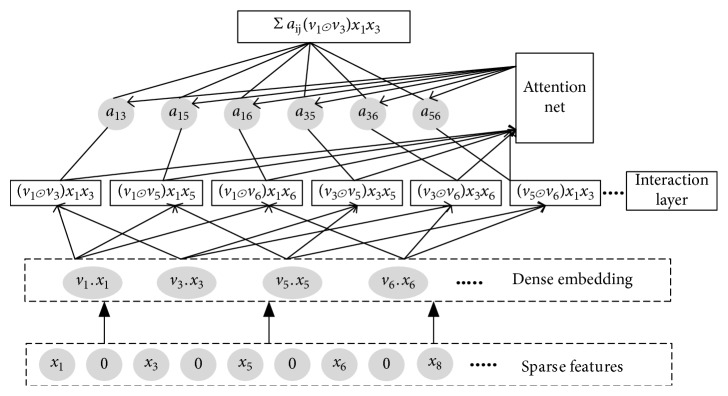
The structure of attentional factorization machines.

Since the attention mechanism has been introduced into neural network modelling, it has been widely used in many tasks. On the basis of FM, Figure 3 shows the neural network structure of attentional factorization machines (AFM). The input layer and the embedding layer are the same as the FM; the input features are represented with sparse features, and each nonzero feature item is embedded in the dense vector. Formally, let the set of nonzero features in the feature vector x be *χ* and the output of the embedding layer be *λ*={*v*_*i*_ *x*_*i*_}_*i*∈*χ*_. In the interaction layer, we can represent the output as a set of vectors:

(10) $$\begin{array}{c} f_{\text{in}}\left(\lambda \right)={\left\{\left(v_{i}⊙v_{j}\right)x_{i}x_{j}\right\}}_{\left(i,j\right)\in R_{x}}, \end{array}$$

where ⊙ denotes the element wise product of two vector and *R*_*x*_={(*i*, *j*)}_*i*∈*χ*,*j*∈*χ*,*j*>*i*_. By defining the interaction layer, we express FM under the neural network architecture. We compress *f*_in_(*λ*) with a sum pooling. Then use the full connection layer to establish it and get the prediction score:

(11) $$\begin{array}{c} \hat{y}=p^{T}\underset{\left(i,j\right)\in R_{x}}{\sum }\left(v_{i}⊙v_{j}\right)x_{i}x_{j}+b, \end{array}$$

where *p* ∈ *ℝ* ^*k*^ denotes the weights and *b* ∈ *ℝ* denotes the bias for the prediction layer.

The attention mechanism has been widely used in many tasks. The idea is to allow different parts to contribute differently when compressing them to a single representation. We use attention mechanisms for feature interaction:

(12) $$\begin{array}{c} f_{\text{att}}\left(f_{\text{in}}\left(\lambda \right)\right)=\underset{\left(i,j\right)\in R_{x}}{\sum }a_{ij}\left(v_{i}⊙v_{j}\right)x_{i}x_{j}, \end{array}$$

where *a*_*ij*_ is the attention score for feature interaction ${\hat{w}}_{ij}$, and it can be interpreted as the importance of ${\hat{w}}_{ij}$ in predicting goals. *a*_*ij*_ can be learned by minimizing the loss function, but the attention scores of interactions that never occur in training data cannot be estimated. In order to solve the generalization problem, we use the multilayer perceptron (MLP) to further parameterize the attention score, which we call the attention network. The input of the attention network is an interaction vector of two features and can encode their interaction information in the embedding space. In general, the attention network is defined as

(13) a′ij=hTRe LUWvi⊙vjxixj+b,aij=expa′ij∑i,j∈Rxexpa′ij,

where *W* ∈ *ℝ* ^*s*×*k*^, *b* ∈ *ℝ* ^*s*^, and  *h* ∈ *ℝ* ^*s*^ are the model parameters and *s* is the hidden layer size of the attention network, which we call the attention factor. Rectifiers are used as the activation function for attention scores and show good performance empirically. The output of the attention layer is a *k*-dimensional vector that compresses all feature interactions in the embedding space by differentiating their importance. We give the overall formulation of attentional factorization machines as

(14) $$\begin{array}{c} y_{\text{AFM}}\left(x\right)=w_{0}+\overset{n}{\underset{i=1}{\sum }}w_{i}x_{i}+p^{T}\overset{n}{\underset{i=1}{\sum }}\overset{n}{\underset{j=i+1}{\sum }}a_{ij}\left(v_{i}⊙v_{j}\right)x_{i}x_{j}, \end{array}$$

where *a*_*ij*_ has been defined in Equation (13).

For the part of the attention network, which is a single-layer MLP, we apply *L*_2_ regularization on the weight matrix *W* to prevent possible overfitting. In other words, the actual objective function we optimize is

(15) $$\begin{array}{c} L={\underset{x\in ϒ}{\sum }\left({\hat{y}}_{\text{AFM}}\left(x\right)-\hat{y}\left(x\right)\right)}^{2}+\lambda {\left\|W\right\|}^{2}, \end{array}$$

where *ϒ* denotes the set of training instances and *λ* controls the regularization strength.

#### 3.3.2. Stacked Autoencoder

**Figure 4:**
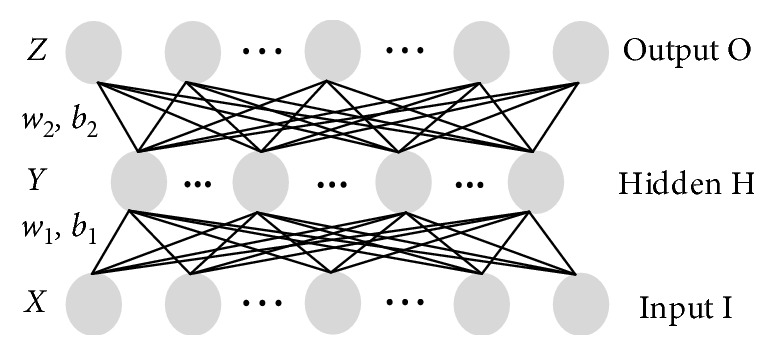
The architecture of the autoencoder.

The autoencoder (AE) [28] is a kind of the neural network model that automatically learns features from data without supervision. It consists of three network layers. The bottom is the input layer I, the middle of the hidden layer H, and the output layer O or reconstruction layer. The autoencoder architecture is shown in Figure 4. In Figure 4, *w* is the connection weight of the two layers and *b* is the bias. In the input layer and hidden layer, the AE model will convert input data to each node of the hidden layer. In the hidden layer and the reconstruction layer, the value of the nodes in the hidden layer is reconstructed and the output data are obtained.

**Figure 5:**
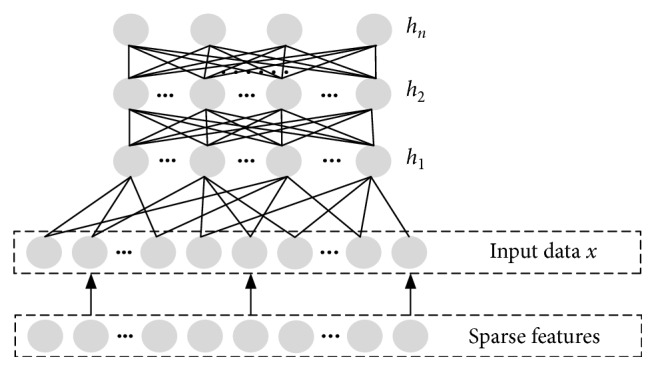
The structure of the stacked autoencoder.

The stacked autoencoder (SAE) [29] is a kind of network that consists of *n* AE stacks from the bottom to the top, as shown in Figure 5. The input data of the bottom AE are *x*. When the training of the bottom AE is finished, the feature of the hidden layer is obtained and can be represented by *h*_1_. Then, *h*_1_ is regarded as the input data of the second AE layer, which is trained and provides the features of the hidden layer and is represented by *h*_2_. This process is repeated until *h*_*n*_ is obtained.

The related definition of the *j*th node in the hidden layer of AE can be described as follows: *s*_*h*_ is the number of nodes in the hidden layer (H) of the AE. *w*_*ji*_^*h*^ is the connection weight between the *j*th node of hidden layer (H) and the *i*th node of input layer (I). *b*_*j*_^*h*^ is the bias of the *j*th node in the hidden layer (H). net_*j*_^*h*^=*b*_*j*_^*h*^+∑_*i*=1_^*s*_*x*_^ *w*_*ji*_^*h*^ *o*_*i*_^*x*^ is the weight sum of the input of the *j*th node in the hidden layer (H). *o*_*j*_^*h*^ is the output value of the *j*th node in the hidden layer (H). The activation function of every neuron node is *σ*(*x*)=1/(1+*e* ^−*x*^).

The output value of the *j*th node in the hidden layer (H) can be represented by the following formula:

(16) $$\begin{array}{c} o_{j}^{h}=f\left({\text{net}}_{j}^{h}\right)=\sigma \left(b_{j}^{h}+\overset{s_{x}}{\underset{i=1}{\sum }}w_{ji}^{h}o_{i}^{x}\right). \end{array}$$

When the feature of the hidden layer (H) is decoded, the feature of the reconstruction layer O is obtained. The output value of the *j*th node in the reconstruction layer O can be represented by the following formula:

(17) $$\begin{array}{c} o_{j}^{o}=g\left({\text{net}}_{j}^{o}\right)=g\left(b_{j}^{o}+\overset{s_{h}}{\underset{i=1}{\sum }}w_{ji}^{o}o_{i}^{h}\right),\\ =\sigma \left(b_{j}^{o}+\overset{s_{h}}{\underset{i=1}{\sum }}w_{ji}^{o}\left(\sigma \left(b_{i}^{h}+\overset{s_{o}}{\underset{k=1}{\sum }}w_{ik}^{h}o_{k}^{x}\right)\right)\right). \end{array}$$

To easily calculate and deduce the formulae, we define the residual error *δ*_*j*_^*l*^ of the *j*th node in the *l*th layer. The residual error *δ*_*j*_^*h*^ of the neuron node of the reconstruction layer can be calculated using the following formula according to the chain rule:

(18) $$\begin{array}{c} \delta_{j}^{h}=\frac{\partial J}{\partial {\text{net}}_{j}^{h}}=\frac{\partial J}{\partial o_{j}^{h}}\frac{\partial o_{j}^{h}}{\partial {\text{net}}_{j}^{h}},\\ =\overset{s_{o}}{\underset{i=1}{\sum }}\left(\frac{\partial J}{\partial {\text{net}}_{i}^{o}}\frac{\partial {\text{net}}_{i}^{o}}{\partial o_{j}^{h}}\right)\cdot \frac{\partial o_{j}^{h}}{\partial {\text{net}}_{j}^{h}},\\ =\left(\overset{s_{o}}{\underset{i=1}{\sum }}\delta_{i}^{o}w_{ji}^{o}\right)\frac{\partial \delta \left({\text{net}}_{j}^{h}\right)}{\partial {\text{net}}_{j}^{h}},\\ =\left(\overset{s_{o}}{\underset{i=1}{\sum }}\delta_{i}^{o}w_{ji}^{o}\right)\delta \left({\text{net}}_{j}^{h}\right)\left(1-\delta \left({\text{net}}_{j}^{h}\right)\right),\\ =\left(\overset{s_{o}}{\underset{i=1}{\sum }}\delta_{i}^{o}w_{ji}^{o}\right)o_{j}^{h}\left(1-o_{j}^{h}\right). \end{array}$$

The parameters *w*_*ji*_^*l*^ and *b*_*j*_^*l*^ can be calculated by formulae (19) and (20):

(19) $$\begin{array}{c} \frac{\partial J}{\partial w_{ji}^{l}}=\frac{\partial J}{\partial {\text{net}}_{j}^{l}}\frac{\partial {\text{net}}_{j}^{l}}{\partial w_{ji}^{l}}=\delta_{j}^{l}\cdot o_{i}^{l-1}, \end{array}$$

(20) $$\begin{array}{c} \frac{\partial J}{\partial b_{j}^{l}}=\delta_{j}^{l}. \end{array}$$

The parameters *w*_*ji*_^*l*^ and *b*_*j*_^*l*^ can be updated as the following formulae, where *ε* is the learning rate:

(21) $$\begin{array}{c} w_{ji}^{l}=w_{ji}^{l}-\beta \frac{\partial J}{\partial w_{ji}^{l}}=w_{ji}^{l}-\beta \cdot \delta_{j}^{l}\cdot o_{i}^{l-1},\\ b_{j}^{l}=b_{j}^{l}-\beta \frac{\partial J}{\partial b_{j}^{l}}=b_{j}^{l}-\beta \cdot \delta_{j}^{l}. \end{array}$$

The SAE is a generative model that is composed of a stack of autoencoders. This method relies on the training algorithm of the autoencoder to initialize the parameters of a stacked autoencoder. Each new layer is stacked on top of the current autoencoder. The process gradually refines the previously learned information and further discovers more complex features. After this, a dense real-value feature vector is generated, which is finally fed into the sigmoid function for CTR prediction:

(22) $$\begin{array}{c} y_{\text{SAE}}=\sigma \left(W^{h+1}\cdot X^{h}+b^{h+1}\right), \end{array}$$

where *W* is the model weight, *b* is the bias, and *h* is the number of hidden layer.

This paper selects the square error as the objective function and adopts the gradient descent [30, 31] to train the parameters, and the objective function can be described by the following formula:

(23) $$\begin{array}{c} J\left(X,O\right)=\frac{1}{2}{\overset{n}{\underset{i=1}{\sum }}\left\|X^{\left(i\right)}-O^{\left(i\right)}\right\|}^{2}. \end{array}$$

#### 3.3.3. ASAE Model

**Figure 6:**
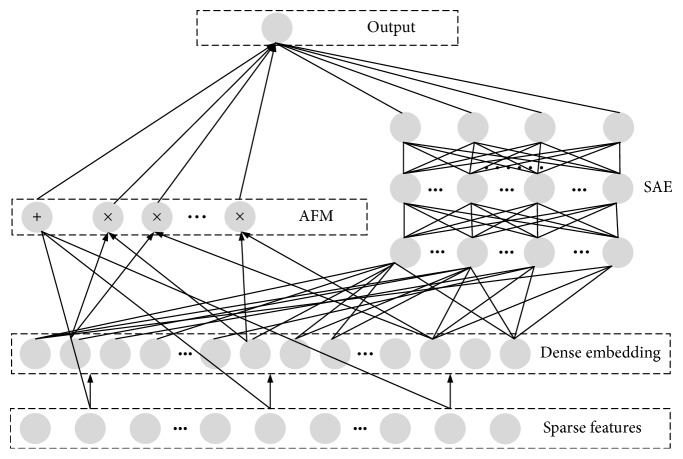
The architecture of ASAE.

The ASAE model consists of two components, AFM component and SAE component, which share the same input. The graphical model of the ASAE model is shown in Figure 6. It is able to learn feature interactions of all orders in an end-to-end manner, without any feature engineering besides raw features. *x*_*i*_ is fed in AFM component to model order-2 feature interactions and distinguish their importance. *x*_*i*_ is fed in SAE component to model high-order feature interactions, and it can generalize better to unseen feature combinations through low-dimensional dense embedding learned for the sparse features. All parameters are trained jointly for the combined prediction model:

(24) $$\begin{array}{c} \hat{y}=\text{sigmoid}\left(y_{\text{AFM}}+y_{\text{SAE}}\right), \end{array}$$

where $\hat{y}\in \left(0,1\right)$ is the predicted CTR, *y*_AFM_ is the output of the AFM component, and *y*_SAE_ is the output of the SAE component.

## 4. Experiments

### 4.1. Datasets

We perform experiments with two publicly accessible datasets: Frappe [32] and SIGKDD Cup2012 track2. The Frappe dataset has been used for context-aware recommendation. It contains 96,215 app usage logs of users under different contexts. Each log contains 8 context variables, including app ID, user ID, city, and daytime. We convert each log into a feature vector with one-hot encoding, resulting in 5,479 features in total. We split dataset into the training set and testing set using a random partition method by the ratio of 8 to 1. The target value of 1 indicates that the user has used the application in context.

The KDD2012 CUP track2 corresponding research question is based on the actual click data information to predict the click rate of the advertisement. The training dataset provided by the competition has a total of 149,639,105 records, and the size of 9.8 GB. In addition to the number of click and the number of displays, the test dataset is consistent with the training dataset, a total of 20,257,594 records, 1.28 GB in size. After data cleaning and data preprocessing, a total of 3.5 million samples were randomly selected from the candidate dataset for the experiment.  Table 1 summarizes the statistics of the final evaluation datasets.

In the KDD2012 CUP track2 dataset, the samples of seven different scale datasets are 150000, 200000, 300000, 500000, 600000, 750000, and 1 million. The training data are grouped randomly, and the final result is the average of all the experimental results to ensure the reliability of the experimental results.

### 4.2. Evaluation Index

We use two evaluation metrics in our experiments: AUC (area under ROC) and Logloss (cross entropy). The curve in AUC usually means the receiver operating characteristic (ROC) [33], which is usually used to measure performance of two-class classifier. The CTR prediction is a classic binary classification method based on whether the advertising is clicked. The value of AUC is usually between [0.5, 1). The larger the value of AUC becomes, the more accurate the advertising CTR prediction is.

### 4.3. Baseline Models

We compare the ASAE model with the following methods that are designed for sparse data prediction:

 
*FM* [34]: FM is successfully applied to the recommended system and user response prediction task. FM explores feature interaction, which is effective on sparse data
 
*FNN* [20]: FNN is a FM-initialized feedforward neural network. It is able to capture high-order latent patterns of multifield categorical data.
 
*CCPM* [19]: convolutional click prediction model (CCPM) is based on convolution neural network. It can extract local-global key features from an input instance with varied elements, which can be implemented for single advertising impression and sequential advertising impression.
 
*Deep cross* [11]: it applies a multilayer residual network on a feature embedding cascade for learning feature interactions. This model is a deep neural network that automatically combines features to produce superior models.
 
*Wide and deep* [35]: this model combines a linear (“wide”) model and a deep model. The deep part is a three-layer MLP that first concatenates feature embedding. The wide part (which is a linear regression model) is subject to design to incorporate cross features.

### 4.4. Analysis of Experimental Results

This section evaluates the ASAE model from two perspectives: (1) discussing the impact of relevant parameters and (2) comparing the ASAE model with five existing prediction models.

#### 4.4.1. Impact of Parameters

**Figure 7:**
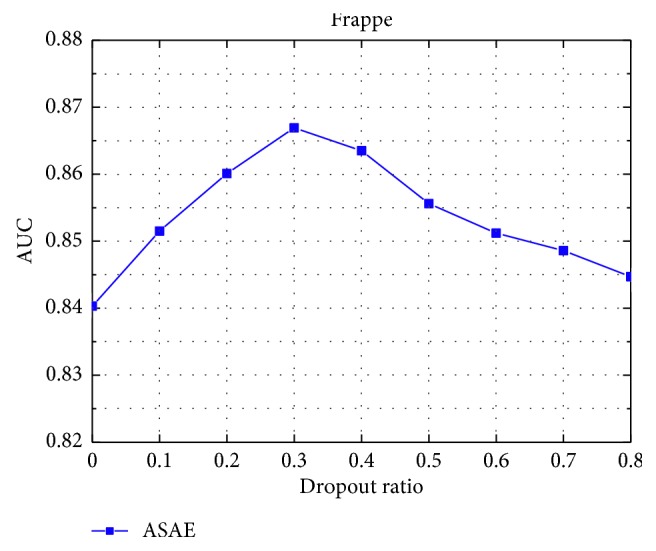
The influence of the dropout ratio.

Dropout [36] refers to the probability that a neuron is kept in the network. Dropout is a regularization technique to compromise the precision and the complexity of the neural network. We set the dropout to be 0.1 to 0.8. As shown in Figure 7, the optimal dropout ratio on Frappe is 0.3. The result shows that adding reasonable randomness to model can strengthen model's robustness.

**Figure 8:**
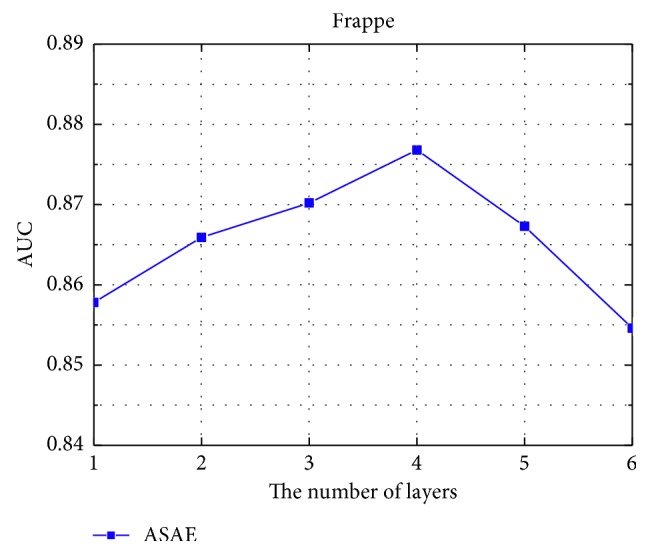
The influence of the number of hidden layers.

The number of network layers *h* in the depth learning phase has a direct effect on the final estimate of the model. Therefore, this paper experimented with parameters to select the better combination of parameters. In the Frappe dataset, as presented in Figure 8, increasing number of hidden layers improves the performance of the models at the beginning. However, with the increasing of the number of hidden layers, the model performance is degraded. This phenomenon is because of overfitting. We can see from Figure 8, the highest AUC value is obtained when the number of hidden layers is 4 in Frappe dataset.

The number of iterations (iter) in the training phase have a direct effect on the final estimation of the model. In the KDD dataset, we used a set of sampled data training models with a data size of 500,000 samples, tested on the test set, and we used it to select the best parameters. While fixing the network layer number (*h*=2, 3, 4, 5, 6) of the model, we can analyze the effect of different iter on the model performance, and the results are shown in Table 2.

**Figure 9:**
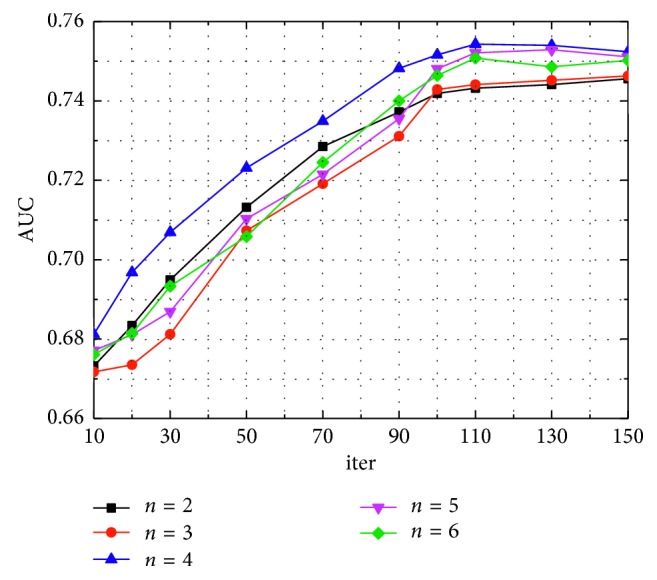
AUC comparison of different iterations and different network layers.

In accordance with Table 2, Figure 9 reflects the AUC change for different network hidden layers *h* and LR model iterations for iter. As seen in Figure 9, when the number of iterations is 90 to 120, the AUC values of several curves stabilized. Therefore, in the comparison experiment, 115 is chosen as the number of iterations for training the prediction model. As shown in Figure 9, the curve fluctuates greatly with the change of iterations, and *h*=4 is relatively stable, so we choose *h*=4.

**Figure 10:**
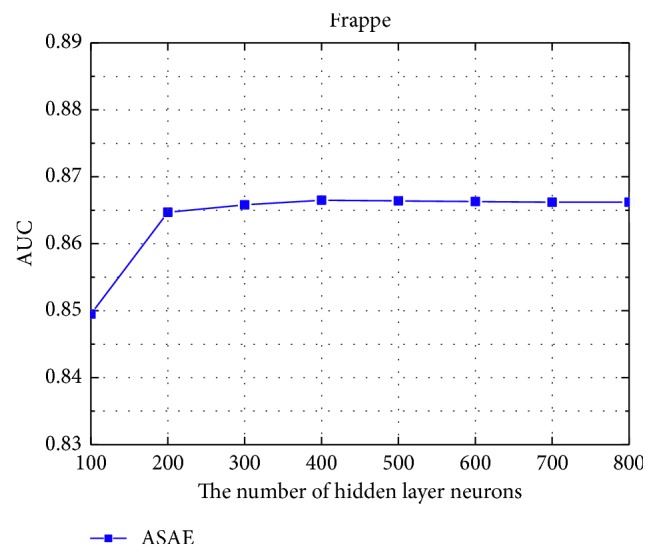
The influence of the number of neurons.

When other factors remain constant, the number of hidden layer units in the ASAE has a huge impact on network performance and the direct cause of the problem is extremely important. However, this figure does not have a general parameter adjustment method in theory. Therefore, in this part, we carry out experiments on the effect of the number of hidden layer neurons. As we can observe from Figure 10, increasing the number of neurons per layer does not always bring benefit. For example, when the number of neurons per layer is increased from 400 to 800, the ASAE performs stably. This is because the complicated model is easy to overfit. In our experiment, 200 to 400 neurons per layer is a good choice.

#### 4.4.2. Performance Comparison

We trained the models on the two datasets and evaluated the estimated results on the same test set. Tables 3 [Table tab4]–5 describe the estimated results for the different methods at different datasets.

Tables 3 [Table tab4]–5 show the overall performance. Compared with the other five methods, the ASAE model showed a better prediction effect. As the data size increased, the accuracy rose and the logloss declined.

 
*FM*: this model was successfully applied to the user response prediction task. It explores feature interaction, which is effective on sparse data. However, this model is limited in mining high-order latent patterns or learning quality feature representations. As shown in Tables 3 [Table tab4]–5, the performance of this model is worst in all comparison models.
 
*FNN*: FNN is a FM-initialized feedforward neural network. The FM pretraining strategy results in some limitations, such as the embedding parameters might be over affected by FM and the efficiency is reduced by the overhead introduced by the pretraining stage. From Tables 3 [Table tab4]–5, we can see that the performance of FNN ranked fifth.
 
*CCPM*: this model is based on convolution neural network for single and sequential advertising impression. However, this model highly relies on feature alignment and is a lack of interpretation. Thus, as shown in Tables 3 [Table tab4]–5, the performance of this model ranked fourth.
 
*Deep cross*: the deep cross is the deepest method that stacks 10 layers above the embedding layer in all compared methods. From Tables 3 [Table tab4]–5, we can see that the performance of this model ranked third due to the problems of overfitting.
 
*Wide and deep*: wide and deep combines a linear model and a deep model. It learns high- and low-order feature interactions. There is a need for expertise feature engineering on the input to the “wide” part. Thus, as shown in Tables 3 [Table tab4]–5, the performance of this model ranked second.
 
*ASAE*: the ASAE model performed best. The reasons are as follows. (1) The input of the model exploits dimension reduction based on decomposition and reduces the sparseness of data. (2) The model takes advantage of the attention mechanism in neural network modelling and improves FM to make feature interactions contribute differently to the prediction. (3) We use improved FM for low-order feature interactions, and stacked autoencoder is used for high-order feature interactions. The model more effectively mines the relationship between features, which can improve the CTR.

## 5. Conclusions

In this paper, based on the search advertising click data, we proposed a sparse feature learning method for advertising data from the perspective of feature learning (DLSAE). We used the reduced dimension method to cluster similar advertisements, queries, and users and established a three-dimensional tensor model for the trial after dimension reduction. Then the low-order approximate tensor was obtained using the Tucker decomposition. Aiming at the highly nonlinear relation between the features, we proposed a hybrid model (ASAE) for advertising CTR estimation based on the stacked autoencoder from the perspective of feature learning. The ASAE model trains a deep component and an AFM component jointly. Performance improved based on these advantages. First, this model does not need any pretraining. Second, it learns both high- and low-order feature interactions, introduces a sharing strategy of feature embedding, and more effectively mines the relationship between features. Last but not least, the proposed model distinguishes the importance of features and makes click-through rate predictions more accurate. More importantly, the importance of feature interactions is automatically learned from the data with any human-domain knowledge. We conducted extensive experiments in two datasets to compare the effectiveness of ASAE with other models. There are two interesting directions for future study. One is exploring a convolutional click prediction model based on CNN for single and sequential advertising impression. And another we are interested in exploring the pooling for recurrent neural networks (RNNs) for sequential data modelling.

## Figures and Tables

**Table 1:** Statistics of the evaluation datasets.

Dataset	Instance	Feature	User	Item
Frappe	276,672	5,479	1,028	5,183
KDD	3,500,000	192,886	127,385	150,672

**Table 2:** The relationship between the number of network layers and iter.

iter	AUC				
	*h*=2	*h*=3	*h*=4	*h*=5	*h*=6
10	0.6732	0.6717	0.6812	0.6771	0.6761
20	0.6834	0.6735	0.6968	0.6811	0.6814
30	0.6949	0.6812	0.7069	0.6869	0.6933
50	0.7132	0.7073	0.7231	0.7103	0.7058
70	0.7285	0.7191	0.7349	0.7215	0.7245
90	0.7372	0.7311	0.7482	0.7355	0.7401
100	0.7419	0.7429	0.7516	0.7481	0.7464
110	0.7432	0.7441	0.7543	0.7521	0.7508
130	0.7441	0.7452	0.7540	0.7529	0.7486
150	0.7456	0.7463	0.7524	0.7511	0.7502

**Table 3:** The performance of AUC and Logloss in different models.

	Frappe					
	FM	FNN	CCPM	Deep cross	Wide and deep	ASAE
AUC	0.7935	0.8012	0.8178	0.8266	0.8293	0.8386
Logloss	0.03842	0.03473	0.03208	0.03127	0.03015	0.02813

**Table 4 tab4:** The AUC performance at different data sizes in KDD dataset.

Data size	AUC					
	FM	FNN	CCPM	Deep cross	Wide and deep	ASAE
150,000	0.6849	0.6941	0.6938	0.7135	0.7193	0.7212
200,000	0.6923	0.7012	0.7027	0.7263	0.7275	0.7355
300,000	0.7033	0.7186	0.7134	0.7397	0.7413	0.7482
500,000	0.7119	0.7235	0.7204	0.7434	0.7486	0.7547
600,000	0.7181	0.7398	0.7291	0.7503	0.7578	0.7672
750,000	0.7255	0.7490	0.7467	0.7549	0.7649	0.7793
1,000,000	0.7315	0.7517	0.7528	0.7652	0.7763	0.7981

**Table 5:** The Logloss performance at different data sizes in KDD dataset.

Data size	Logloss					
	FM	FNN	CCPM	Deep cross	Wide and deep	ASAE
150,000	0.02967	0.02925	0.02941	0.02826	0.02714	0.02733
200,000	0.02898	0.02893	0.02882	0.02793	0.02665	0.02704
300,000	0.02886	0.02872	0.02876	0.02768	0.02657	0.02658
500,000	0.02875	0.02864	0.02872	0.02759	0.02652	0.02639
600,000	0.02783	0.02715	0.02713	0.02674	0.02645	0.02630
750,000	0.02752	0.02739	0.02728	0.02631	0.02627	0.02543
1,000,000	0.02677	0.02642	0.02634	0.02628	0.02606	0.02501

## Data Availability

The data used to support the findings of this study are available from the corresponding author upon request.
